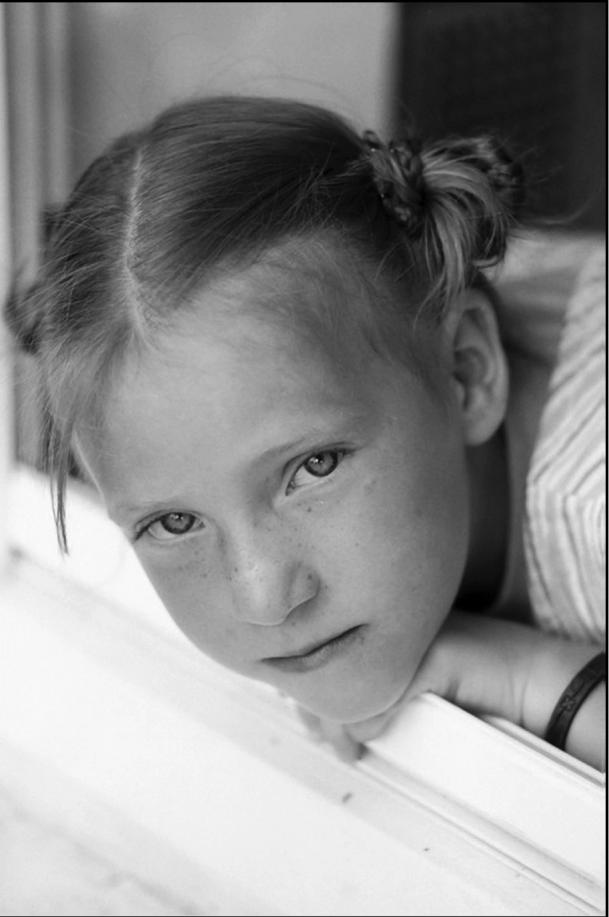# Open House: The Ethics of Studying Children at Home

**DOI:** 10.1289/ehp.114-a168

**Published:** 2006-03

**Authors:** Ernie Hood

Home is where the heart is, but for children, home all too often can be where the danger is—danger of exposure to lead, asthma triggers, pesticides, safety hazards, and other potential sources of harm. Those risks are disproportionately common among poor and minority children, whose families more often lack access to decent, affordable housing.

In recent years, environmental health scientists have increasingly sought to identify and ameliorate risk factors affecting such children. Research into housing-related health hazards involving children has proven to be an area of investigation both rich in potential for discovery of effective intervention methods and fraught with opportunities for ethical lapses. A recent report by the National Academies now provides researchers and their sponsors clear guidelines to avoid ethical pitfalls while aggressively pursuing new and beneficial knowledge.

A 2001 decision by the Maryland Court of Appeals cast a spotlight on the ethics issue. In the case *Grimes v. Kennedy Krieger Institute*, two mothers sued researchers, claiming they had placed child subjects in a lead abatement study at an unacceptable level of risk. In determining that the case should proceed to trial, the court included comments in which it scolded the research community for what it perceived to be shortcomings in the approach to conducting research involving low-income populations, particularly with regard to obtaining truly informed consent from the parents of children participating in such studies.

Although *Grimes v. Kennedy Krieger Institute* was eventually settled out of court, the appeals court comments threw the field into a state of confusion as its ethical underpinnings were called into question. Ultimately, this uncertainty led the major federal funders of this type of research—HUD, the CDC, and the EPA—to ask the National Academies to appoint a committee to examine the issues in detail and recommend procedural and policy changes to clarify best practices and resolve ethical dilemmas.

The Committee on Ethical Issues in Housing-Related Health Hazard Research Involving Children, Youth, and Families, a project of the National Research Council and the Institute of Medicine, was carefully chosen to represent a variety of stakeholders in this research enterprise. “We were diverse in terms of background and professional training,” says committee chairman Bernard Lo, a professor of medicine and director of the Program in Medical Ethics at the University of California, San Francisco. “We had scientists who do housing research, and we had several members who were very familiar with low-income housing and were advocates for people in low-income housing. Those are the sorts of viewpoints that needed to be included, supported, and recorded.”

## Peeking into Scary Closets

A variety of ethical concerns coalesce to make housing-related health hazard research particularly challenging. It takes place in the home, with an inherent invasion of privacy. While in the home, researchers may notice hazardous conditions other than those under study—what is their responsibility in such a situation? The community may have expectations of and desires for ameliorative research outcomes that are vastly different from those of the investigators. Many health hazards (such as lead-based paint) occur disproportionately in poor-quality housing occupied by low-income, often ethnic minority families, so children in these families are the most likely candidates for study. This can lead to concerns about exploiting vulnerable groups of subjects for research that ultimately benefits others. Parents in these families are often poorly educated; this, combined with the typical complexity of informed consent forms, makes it difficult for them to provide meaningful informed consent for their children’s participation. Also, inappropriate financial and other incentives may unduly influence parents’ decisions regarding their children’s participation.

The committee met five times over the course of 18 months to examine these issues. The panelists heard presentations from committee members and external experts, including parents, community leaders, researchers, government officials, and specialists in law and ethics. “Through those presentations, as well as their review of the literature and their deliberations, the committee came up with their consensus view on what ought to be done in this area,” says Mary Ellen O’Connell, a National Academies staff officer who was the committee’s study director. After the group’s initial draft was reviewed by an external slate of reviewers with similar expertise and revised based upon their comments, the final report, *Ethical Considerations for Research on Housing-Related Health Hazards Involving Children*, was released on 19 Septem-ber 2005. Lo says the committee unanimously supported all of the recommendations put forth in the report.

The report addresses specific recommendations to the three main audiences in need of guidance: researchers themselves, research institutions and institutional review boards, and the federal government and other research sponsors. The recommendations all revolve around two guiding themes: the need to involve community representatives at all stages of the research, from inception to follow-up, and the need to strengthen the process of informed consent so that parents fully understand the essential features of the research study. The report strikes an elegant balance, clarifying what have been ethical gray areas while still facilitating and encouraging housing-related health hazard research designed to improve the lives of the most vulnerable children and families.

## Building a Firm Foundation

One key recommendation made by the committee is that all federal agencies sponsoring housing-related health hazard research should formally adopt the federal regulations addressing human subject research participation, particularly Subpart D of 45 CFR 46, which provides additional protections specifically for child participants. The CDC and the NIEHS, as agencies of the Department of Health and Human Services, are already governed by Subpart D. According to Lo, the EPA has committed to formally adopting it, while HUD has responded that it will follow the regulations and require their protections in their projects, but does not plan to make Subpart D part of its official policy, at least for now.

According to committee member Alan Fleischman, a senior advisor at the New York Academy of Medicine, the panel was particularly impressed with the amount of data supporting the value of community engagement in environmental health research—so-called community-based participatory research (CBPR). “It actually results in better, more focused research,” he says. “There is increasing evidence that engaging communities actually makes the research more powerful, more important, and more valid, and the potential to develop advocacy approaches to do public health intervention and change is more effective.” Further, subjects are better protected in that community involvement can act as kind of a buffer, ensuring that potential risks and benefits are well characterized and defined, and that there is far less potential for even inadvertent exploitation.

The committee recognized that in the real world the reforms they were recommending would translate into more time and more money being required for housing-related health hazard research projects. The panel addressed this reality by recommending that research sponsors provide the additional funding and extended timelines necessary to support expanded community participation. The report also suggests that “researchers need to develop ongoing partnerships with their communities, which is of course complex, and takes time and effort,” says Fleischman. But in the long run, he adds, “individual research projects may not be slowed down, if in fact those projects are part of a portfolio of research being done with relationship to the community as partners.”

## Putting Out the Welcome Mat

By most accounts, the report has been received quite warmly. “This report was particularly important to us,” says Rebecca Morley, executive director of the nonprofit National Center for Healthy Housing, which both sponsors and conducts research. “In order to do our research, we were looking forward to having very clear guidelines, because what we were finding is that the current [oversight approaches] had the ironic and perverse effect of discouraging the study of the most serious health hazards, and prompted researchers to shy away from studies that focus on communities at the highest risk, for fear of being seen as callous or discriminatory,” she says.

According to Lo, feedback from HUD, the EPA, and the CDC has been positive. “They’ve already adopted some of our recommendations,” he says. “They responded favorably, and they are obviously looking at the recommendations carefully, and hopefully they’ll encourage all of their researchers to adopt them.”

As an institute that has pioneered CBPR—in fact, often requiring community involvement in the research process—the NIEHS has also welcomed the refinements offered in the report. Children’s environmental health program administrator Kimberly Gray says that although the institute already practices much of what the report preaches, it’s good to get a wider outside perspective on the issues involved.

O’Connell thinks the tone of the responses to the report she’s been hearing bodes well for acceptance and active adoption of its recommendations. She says, “People have been asking me ‘how do we do this?’ rather than ‘why should we do this?’”

## Rooms with a Long View

Of course, a committee report, however thorough and well-intentioned, cannot guarantee that ethical lapses will not still occur, that researchers might not still hesitate to pursue housing-related health hazard research projects for fear of litigation, or that its recommendations and suggestions will be universally followed. But committee members are optimistic that the report will be viewed as a milestone in efforts to support and encourage such research while putting it on a more solid ethical footing.

“We believe that these are feasible recommendations that are well within the ability of researchers and sponsors to carry out, and that they will improve trust within the communities that they serve, and also strengthen parents’ understanding of what this research is all about,” says Fleischman.

Morley is taking more of a wait-and-see approach. “The proof will be in the pudding in implementation,” she says. “As researchers actually start to apply this, I think we will see the practicality of the recommendations, and whether [the report] has delved into sufficient detail to enable researchers to operate with a clear conscience and also give participants peace of mind.”

## Figures and Tables

**Figure:**
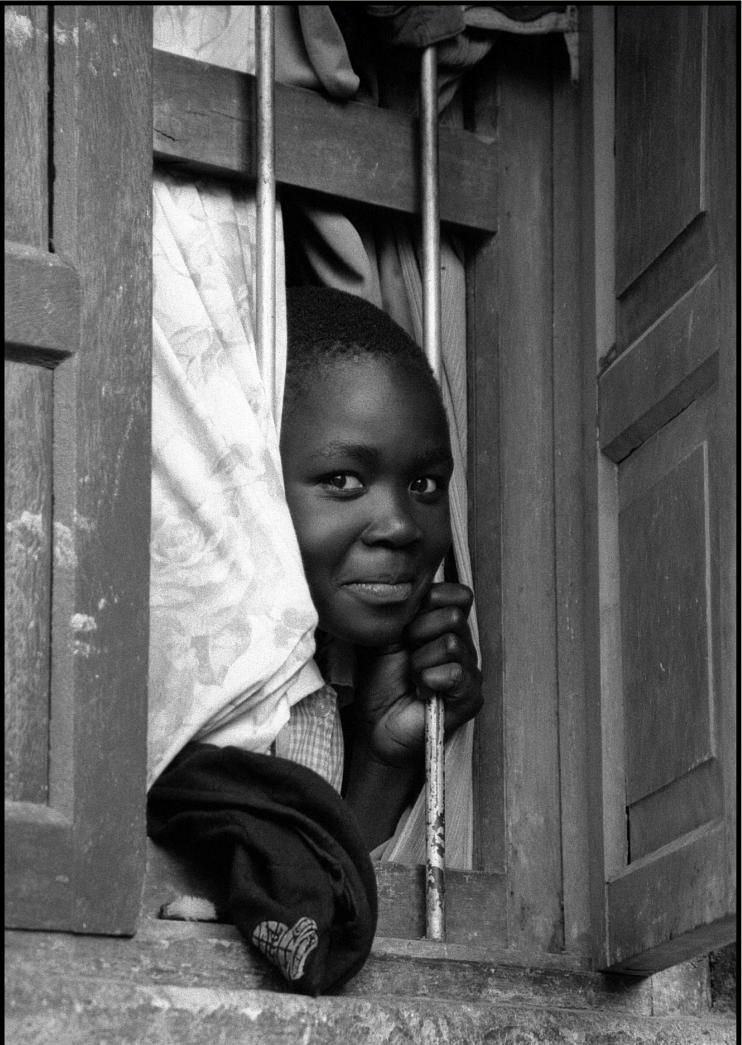


**Figure:**